# Progressing Cross‐Sector Collaboration for People With Eating Disorders and Higher Weight: Priority Actions From an Expert Roundtable Using a Modified Nominal Group Technique

**DOI:** 10.5694/mja2.70235

**Published:** 2026-07-01

**Authors:** Hiba Jebeile, Leah Brennan, Tracy Burrows, Xochitl de la Piedad Garcia, Angelique F. Ralph, Supreet Saluja, Evan Atlantis, Sarah P. Garnett, Carmel J. Harrison, Eve T. House, Natalie B. Lister, Lisa Moran, Milan K. Piya, Elizabeth Rieger, Evelyn Smith, Phillipa Hay, Sarah Trobe

**Affiliations:** ^1^ Faculty of Medicine and Health Sydney Medical School, The University of Sydney Westmead New South Wales Australia; ^2^ Charles Perkins Centre The University of Sydney Sydney New South Wales Australia; ^3^ School of Psychology and Public Health, La Trobe University Wodonga Victoria Australia; ^4^ Centre for Eating, Weight and Body Image Melbourne Victoria Australia; ^5^ College of Health, Medicine and Wellbeing, Hunter Medical Research Institute, The University of Newcastle Australia Newcastle New South Wales Australia; ^6^ School of Behavioural and Health Sciences, Australian Catholic University Fitzroy Victoria Australia; ^7^ Nepean Blue Mountains Family Metabolic Health Service Nepean Blue Mountains Local Health District Penrith New South Wales Australia; ^8^ School of Health Sciences, Western Sydney University Penrith New South Wales Australia; ^9^ Translational Health Research Institute, Western Sydney University Penrith New South Wales Australia; ^10^ BodyMatters Australasia Dickson Australian Capital Territory Australia; ^11^ Discipline of Psychology University of Canberra Canberra Australian Capital Territory Australia; ^12^ Monash Centre for Health Research & Implementation Monash University Melbourne Victoria Australia; ^13^ Macarthur Diabetes, Endocrinology and Metabolism Service Camden and Campbelltown Hospitals, South Western Sydney Local Health District Campbelltown New South Wales Australia; ^14^ School of Medicine and Psychology, Australian National University Canberra Australian Capital Territory Australia; ^15^ School of Psychology, Western Sydney University Penrith New South Wales Australia; ^16^ Mental Health Services Camden and Campbelltown Hospitals, South Western Sydney Local Health District Campbelltown New South Wales Australia; ^17^ National Eating Disorder Collaboration Sydney New South Wales Australia

**Keywords:** feeding and eating disorders, obesity, paediatric obesity

## Abstract

**Introduction:**

Eating disorders are more prevalent in people with higher weight than those with low weight. However, contention between the fields of obesity and eating disorders has prevented meaningful progress in research, prevention, identification and coordinated clinical services for people with co‐occurring conditions. In Australia, public health approaches and provision of treatment services for people with eating disorders and clinical obesity are siloed, often resulting in contradictory messaging. To address this, a roundtable meeting was held in November 2024 in Sydney, Australia, with 28 experts in one or both of these fields, including researchers, clinicians and service leaders working across paediatric and adult care, and individuals with lived experience. Guided by the National Eating Disorders Collaboration stepped system of care framework, participants identified key challenges and possible solutions, and established five priority actions.

**Main Recommendations:**

The priority actions across sectors are: *Health Campaigns* focused on raising awareness of eating disorders at higher weight, using appropriate language and reducing weight stigma; improved *Screening and Assessment* using standardised protocols across healthcare settings; supporting *Primary Healthcare* and improving the use of Medicare items; *Tailored Treatment Pathways* including integrated care models; and building *Workforce Capacity* to upskill professionals to provide safe, person‐centred care.

**Changes in Management as a Result of the Statement:**

These actions aim to promote improved cross‐sector collaboration and effective, safe, coordinated and integrated approaches to prevention, identification and treatment across the fields of obesity and eating disorders. They address the complex medical and psychological needs of those with co‐occurring eating disorders and higher weight or clinical obesity through a skilled workforce and improved access to care. Effective integration, collaboration and coordination across services is essential for long‐term recovery support.

## 1 Background

Clinical obesity and eating disorders are both prevalent health conditions driven by a complex interaction of biological, genetic, psychological, social and environmental factors [1, 2, 3, 4]. Although diagnostically independent [5, 6], they co‐occur across the lifespan with increasing prevalence [7, 8, 9, 10]. A long‐standing divide between the fields of obesity and eating disorders has created significant barriers to meaningful progress in research, prevention, identification and provision of clinical services for people affected by both conditions [11, 12, 13, 14, 15, 16, 17]. Clinical eating disorders (e.g., binge eating disorder, atypical anorexia nervosa) and subclinical disordered eating (e.g., binge eating, loss of control, emotional eating, extreme restriction) are prevalent in people with higher weight (Box 1) [8, 9, 10]. While research has focused on binge eating disorder in people with higher weight, atypical anorexia nervosa (i.e., all symptoms of anorexia nervosa except low weight) is also prevalent in this group [8, 18]. Despite this overlap, obesity and eating disorder treatment services are poorly integrated. Clinical obesity services seldom provide psychological support to assess for eating disorders or disordered eating [19]. Similarly, clinicians providing eating disorder treatment often don't assess adiposity‐related health and this is not addressed in traditional eating disorder interventions. Beyond assessment, contradictory messages regarding clinical care can cause confusion and harm among people with this comorbidity. For example, while obesity clinicians typically recommend weight management as part of a comprehensive intervention, eating disorder clinicians often perceive weight loss interventions as ineffective, unsafe and/or unnecessary. This siloed approach [20] has resulted in a lack of communication across fields, and contradictory public health and treatment advice. Consequently, people living with eating disorders and higher weight are stigmatised and not offered or provided with effective treatment. There is a need for greater collaboration and exchange of knowledge and clinical expertise across fields. This article summarises findings of an expert roundtable designed to identify shared goals and key priority areas for action across the fields of obesity and eating disorders in Australia.

BOX 1 **Weight‐related language.**

The language used to describe body weight is emotionally laden and should be informed by lived experience. In one‐on‐one interactions (including clinically), permission should be sought before discussing body weight, and the language chosen should be informed by the preferences of the individual concerned [21]. In this manuscript, person‐first language is used, and in most instances, the term ‘higher weight’ is used as a more neutral preferred term by people with lived experience [21, 22, 23, 24]. The term ‘clinical obesity’ is also used, particularly when discussing obesity treatment. The 2025 Lancet Commission on the definition and diagnostic criteria of clinical obesity refers to classifications of adiposity‐related risk as ‘preclinical obesity’ and ‘clinical obesity’. Clinical obesity refers to a specific subset of people living with higher weight who are experiencing disturbances to organ or tissue function or limitations to their participation in activities of daily living attributable to excess adiposity [5]. People with clinical obesity have clinical treatment needs that should be considered in conjunction with any identified need for eating disorder treatment [5].

## 2 Methods

The Eating Disorder In weight‐related Therapy (EDIT) Collaboration [25] and the National Eating Disorders Collaboration (NEDC) hosted a roundtable meeting, using a modified nominal group technique, in November 2024 in Sydney, Australia. The methods are described in the Supporting Information. In brief, participants (*N* = 28) were purposively selected to ensure equal and diverse representation of researchers, clinicians, service leaders and people with lived experience across fields. The NEDC stepped system of care framework [26] (Supporting Information) was used to identify key challenges and future directions to advance the fields across the life course.

**FIGURE 1:**
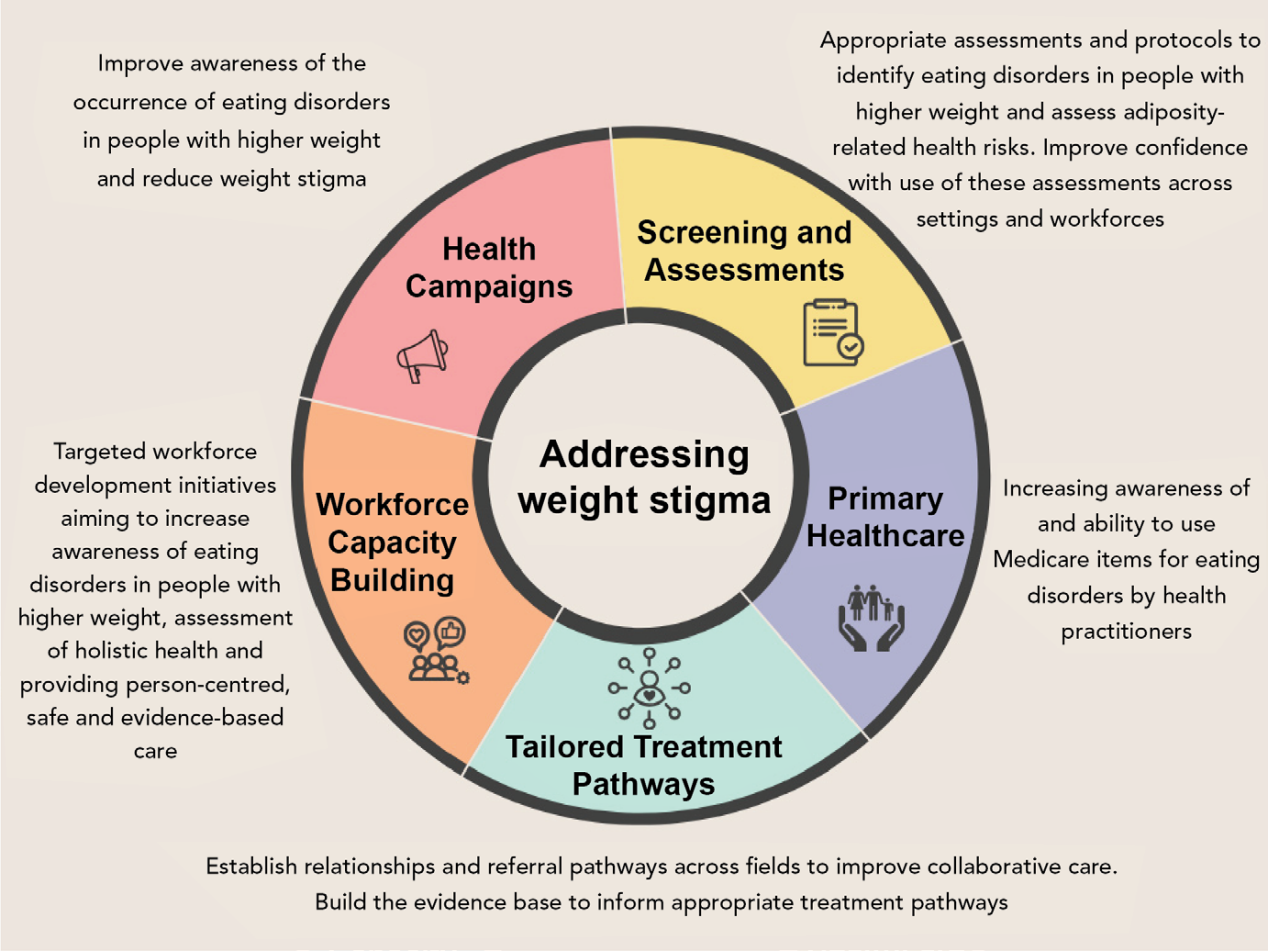
Priority actions arising from the eating disorders and higher weight roundtable, November 2024.

The roundtable included invited presentations to set the scene on the intersection of eating disorders and higher weight and to share the lived experience. For each component of the NEDC stepped system of care (prevention, identification, initial response, treatment, and psychosocial and recovery support), participants identified current barriers that affect outcomes for people with eating disorders and higher weight. These were synthesised and summarised into common themes. For each barrier, participants then identified existing programs and research addressing these, and opportunities for future initiatives. Finally, participants were asked to identify five priority areas of action from those identified (Table S1). These were tallied and discussed to reach agreement (Figure 1).

## 3 Recommendations

### 3.1 Cross‐Cutting Themes

Considerations of weight stigma, cultural competence, gender, sexual diversity and neurodiversity were central to each priority action. Applying an intersectional lens to proposed solutions and actions facilitates consideration of the complexity of a person's experiences. Within this article, we focus on weight stigma—that is, the negative beliefs, attitudes and discrimination faced by people with higher weight because of their body size—as this may be experienced by all people with higher weight. Weight stigma is prevalent across settings (media, schools, workplace, healthcare) and is an influential factor across the system of care. In healthcare, this can present at the structural level (e.g., policies, referral criteria and guidelines that are not inclusive of size diversity), in the built environment (e.g., small chairs, equipment and hospital beds) [27] and interpersonally (e.g., negative language, biased healthcare provider attitudes) leading to reduced trust and healthcare avoidance [28, 29]. At an intrapersonal level, internalised weight bias involves the acceptance of negative attitudes/beliefs about people with higher weight and the application of these to the self, leading to self‐devaluation and is associated with negative biological and psychological outcomes, including disordered eating, weight gain and poor biopsychosocial health [30, 31, 32, 33, 34]. Weight stigma may impede the identification of disordered eating behaviour and cognition in people with higher weight [29, 35]. Research is needed to understand these effects, how to overcome them and the role of interventions targeting reduced weight stigma [36].

As part of safe and effective treatment, it is essential that health professionals are aware of and address their own negative weight‐related attitudes and beliefs, and listen openly to a person's concerns. Some health professionals may avoid any discussion of adiposity‐related health risk due to fear of being stigmatising. Acknowledgement and minimisation of weight stigma in healthcare does not require avoidance of weight‐related conversations, rather guidance on how to have these conversations in a respectful and sensitive way, consistent with person‐centred care. Health professionals also need to be aware of less recognised forms of stigma directed towards people who opt to seek weight‐loss interventions and towards those who choose not to lose weight. Several resources which aim to address and minimise weight stigma have been developed [37, 38, 39, 40], and further awareness and dissemination of these is needed.

### 3.2 Priority Areas for Action

#### 3.2.1 Health Campaigns

Eating disorder prevention focuses on improving body image and reducing stigma for people of all body sizes, and discouraging active attempts at weight loss [41]. In contrast, obesity prevention has highlighted that higher weight is associated with health risks, and that maintaining a body weight within a ‘healthy’ weight range is important. High‐quality data on the impact of each of these prevention approaches on holistic health, and potential negative impacts, are scarce, and these conflicting messages are confusing for consumers. Solutions focused on increased awareness of the co‐occurrence of eating disorders and higher weight, shared risk factors and addressing weight stigma are needed.

Schools were identified as a setting for early intervention to provide preventative health information, improve health literacy and address misinformation. Providing education on nutrition and physical activity is a core part of the Australian school curriculum. However, how this is delivered varies based on the skills and experience of educators. Messaging intended to promote healthy eating and reduce intake of ultra‐processed foods, for example, can be delivered in a way that contradicts eating disorder prevention by framing foods as ‘good’ versus ‘bad’. There are effective evidence‐based programs that focus on improving body image alongside health behaviour [42], but these are not yet universally available. Similarly, public health campaigns can target both eating disorder and obesity prevention through promoting healthful eating behaviours, encouraging enjoyable and safe physical activity, supporting body appreciation and reducing the focus on individual responsibility for body size and shape [41].

Priority actions include co‐designed and evidence‐based health campaigns to raise awareness of the incidence of the spectrum of eating disorders, in individuals with higher weight, and ensuring safe and consistent messaging across fields and settings.

#### 3.2.2 Screening and Assessment

Effective treatment of eating disorders requires the availability, accessibility and timely use of sensitive and specific screening and assessment tools to identify eating disorders in people with higher weight. This is important as early intervention of eating disorders is associated with positive impacts on prognostic outcomes and overall disease burden [43]. A key issue is that many assessment tools were informed by understanding of low‐weight eating disorders and restraint theory and/or focus on specific eating disorders (i.e., anorexia nervosa and bulimia nervosa). While self‐report questionnaires have been developed to screen for eating disorders and prompt further assessment [44, 45], their validity in higher weight individuals, especially for those seeking treatment for clinical obesity [5], remains to be examined [38, 46]. There are currently no assessments developed and tested for the detection of the spectrum of eating disorders in individuals with higher weight [46]. In addition, medical and allied health providers lack confidence with existing measures [20], and this prevents consistent implementation despite guideline recommendations [38, 47, 48].

Similarly, clinicians providing eating disorder assessment and treatment for people with higher weight may not assess adiposity‐related health risk. Conversations about body size and risk are sensitive [21, 22] and clinicians may be cautious of being stigmatising and exacerbating eating disorder symptoms. Clinicians may also lack knowledge and/or confidence with use of existing tools such as the Edmonton Obesity Staging System‐2 (EOSS‐2) Risk Tool, which aims to stage severity of metabolic, mental and functional health problems associated with clinical obesity [37, 49, 50].

Priority actions to improve assessment of holistic health for people with eating disorders and higher weight, include: (i) determining best practice screening and assessment approaches—for eating disorders, this may require modifying and evaluating existing assessments; and (ii) implementing screening and assessment protocols across settings (e.g., primary care, mental health, obesity clinics, bariatric surgery), with appropriate training.

#### 3.2.3 Primary Healthcare, Including Medicare

Integration of care for eating disorders and higher weight needs to occur across specialist, non‐specialist and primary care settings. This is critical given the limited capacity of specialist services, and the heightened health needs in this population. Health infrastructure and funding must support appropriate screening, assessment and intervention. Treatment for eating disorders and clinical obesity can be delivered through community‐based care under Medicare, but the system lacks clear guidance on the optimal use of existing Medicare items for individuals with eating disorders and higher weight.

Introduced in 2019, Eating Disorders Treatment and Management Plans (EDPs) support eating disorder treatment. A 2024 evaluation of EDPs suggested that health practitioners may have more difficulty determining eligibility of individuals with eating disorders that are not characterised by low weight [51]. For individuals with an eating disorder other than anorexia nervosa, several additional criteria are needed to meet EDP eligibility (e.g., occurrence of medical complications due to the eating disorder). However, individuals with eating disorders and higher weight may not be recognised as eligible under this criterion as medical risk may be attributed to, or underestimated because of, weight status. For example, binge eating disorder is associated with metabolic syndrome [52, 53] and the significant medical risks associated with anorexia nervosa are seen in people with higher body weight (i.e., atypical anorexia nervosa) [54]. These health consequences are typically either overlooked or considered a consequence of higher weight rather than the eating disorder, so the criterion is not considered met. Further, people who have had metabolic and bariatric surgery may present with loss‐of‐control eating, but the amount of food is not ‘objectively large’ (as required for an eating disorder diagnosis) due to the anatomical changes associated with surgery. While the size of the binge is smaller post‐surgery, loss‐of‐control eating continues and is associated with increased eating disorder psychopathology and impairment, requiring specialised eating disorder treatment [55]. In addition, the Eating Disorder Examination Questionnaire likely underestimates eating disorder pathology in those with non‐restrictive eating disorders and for those post‐surgery [56, 57]; thus, some people in these groups do not meet the EDP clinical cut‐off. Consequently, these individuals may not be considered eligible for an EDP despite presenting with complex psychological and nutritional needs requiring the multidisciplinary care that the EDP offers.

Individuals who are not eligible for an EDP may access mental healthcare under a Mental Health Treatment Plan (MHTP), through the Better Access initiative, and allied healthcare under a GP Chronic Condition Management Plan (GPCCMP). GPCCMPs offer access to several allied health professionals which may be required for an eating disorder and/or clinical obesity. However, there is often confusion as to whether clinical obesity can be treated under the plan because obesity was not consistently recognised as a chronic disease prior to *The Lancet Diabetes and Endocrinology* Commission on the definition and diagnostic criteria of clinical obesity [5]. In addition, GPCCMPs offer fewer visits and lower Medicare rebates than MHTPs and EDPs, contributing to high out‐of‐pocket cost as a barrier to care. Indeed, most Medicare‐subsidised services attract gap fees, making care inaccessible for those without the financial means to pay. There is a need to increase MHTP and GPCCMP session numbers, raise rebates and introduce Medicare items for extended medical consultations, case management and interprofessional case conferencing.

Priority actions include: (i) improving the use of existing Medicare items and supporting health professionals to effectively navigate these; (ii) expanding Medicare items to recognise the complexity and chronicity of eating disorders and clinical obesity, and to enable comprehensive, interdisciplinary and long‐term care; and (iii) exploring funding models that reduce reliance on out‐of‐pocket costs.

#### 3.2.4 Tailored Treatment Pathways

In Australia, treatment pathways and services for eating disorders and clinical obesity are siloed. Guidelines on the treatment of eating disorders in people with higher weight were published in 2022 [38], and updated Australian guidelines for the treatment of overweight and obesity are in development. Coordinated implementation across fields will be essential to improve outcomes. Several gaps in relation to the accessibility and provision of evidence‐based care across settings were discussed. Access to specialist services for both eating disorders and clinical obesity is rare. Support for preclinical or clinical obesity and related complications is often managed through primary healthcare services; there are few multidisciplinary tertiary clinical obesity services across Australia [19, 58], and only 5.5% of bariatric surgery occurs in the public system [59]. With the introduction of the Australia and New Zealand Academy for Eating Disorders Eating Disorder Credential [60], availability of evidence‐based eating disorder treatment services has expanded, though for the most part, these are privately funded. General practitioners are well placed to coordinate care in the community, but lack of awareness and expertise on eating disorders, particularly in people with higher weight, poses challenges for clinical care. Establishing relationships and collaborative services across eating disorders and obesity is essential to support person‐centred care over the long term.

Stepped care across primary, secondary and tertiary services, with shared goals between these, is essential to prevent mixed messages and contradictory interventions for the individual. Significant overlap between evidence‐based treatments for clinical obesity and eating disorders exists. Psychological and behavioural interventions for both involve regular planned eating, problem solving skills, stimulus control, increasing enjoyable movement or exercise, improving body image and challenging weight stigma [61, 62]. Guidance is needed on when to prioritise treatment of one condition over the other, and when providing co‐treatment is appropriate, with individuals informed, empowered and equipped to be involved in decision making consistent with person‐centred care.

Further research is needed on the effect of obesity treatment on eating disorder outcomes, eating disorder treatment on metabolic outcomes, and how we can move towards personalised medicine for individuals with both conditions [63]. Co‐treatment models for people with eating disorders and clinical obesity—informed by shared neurobiology, building on current evidence and propelled by emerging integrated treatments—are needed [2, 61, 64, 65, 66, 67, 68].

Priority actions include: (i) establishing relationships and referral pathways across fields from primary through to specialist care; and (ii) building the evidence base to inform collaborative, safe and effective person‐centred and personalised treatment pathways and models of care that address the physical and mental health needs of each person.

#### 3.2.5 Workforce Capacity Building

Many healthcare professionals and researchers have expertise in either eating disorders or obesity. However, since most people with eating disorders have higher weight [8], and many people presenting for obesity treatment report disordered eating [10], they would clearly benefit from expertise drawn from both fields [17, 69]. Workforce development is fundamental to delivering high‐quality integrated care. Building capacity across workforces—including primary care, specialist services, mental health, nursing, allied health and tertiary education—and ensuring consistent, evidence‐based messaging across providers is crucial.

There is currently insufficient provider training that bridges eating disorders and clinical obesity, and this has led to inconsistent messages, fragmented care and missed opportunities for early identification. There are specific workforce gaps relating to sensitive and appropriate care for high‐risk groups such as children and adolescents, those transitioning to young adulthood, people with a history of trauma, women across the life course (fertility, pregnancy, motherhood, perimenopause, menopause), First Nations peoples, culturally and linguistically diverse groups, and people living with one or multiple chronic health conditions (e.g., diabetes). Addressing this requires embedding weight‐inclusive content on eating disorders, weight stigma and clinical obesity into medical and allied health professional training programs, professional bodies and societies, and across specialty areas commonly involved in the care of people with higher weight (e.g., cardiology, hepatology).

Creating a safe and supportive environment for health professionals to openly discuss, ask questions and build understanding of the shared neurobiology, identification of co‐occurrence and personalised treatment pathways is essential for effective workforce development. Expanding and integrating the peer workforce is also key, particularly in supporting individuals with lived experience of both conditions. Dissemination of existing resources and care navigation tools, supported by partnerships with the community sector, will further enhance the workforce's capacity to deliver holistic, integrated care.

Priority actions include: (i) developing and disseminating targeted workforce initiatives aimed at increasing awareness, identification and referrals for people with eating disorders and higher weight; and (ii) upskilling health professionals to have expertise in both eating disorders and clinical obesity, to provide respectful integrated, person‐centred, safe and evidence‐based treatment.

## 4 Conclusions

Transformation of the system of care for eating disorders and higher weight is needed to break down silos and achieve integrated prevention and treatment approaches across the life course. Across the health system, Eating Disorder Safe Principles (policy and practice framework aimed at embedding eating disorder prevention and harm minimisation across settings; Supporting Information) should be adopted by services to ensure physically safe and welcoming environments with appropriate equipment [39] and consideration of language preferences (Box 1). Primary care is central to early identification of eating disorders and clinical obesity and coordination of care. Evidence‐based and accessible referral pathways are needed to ensure people can seamlessly access the respectful, safe and effective multidisciplinary care they need without falling through the gaps.

The five priority actions that we have described are intended to support collaborative and coordinated prevention efforts, greater consideration of eating disorders in the treatment of people with clinical obesity and greater consideration of adiposity‐related health risks in the treatment of people with eating disorders. Fundamental to advancing the much‐needed collaboration across the eating disorders and obesity fields will be the cultivation of safe and productive discourse between these fields, grounded in respect, humility and openness to challenging our assumptions as we seek to effectively and compassionately support those currently caught at the intersection of these fields.

## Author Contributions

Project administration: Hiba Jebeile and Sarah Trobe. Writing – original draft: Hiba Jebeile, Leah Brennan, Tracy Burrows, Xochitl de la Piedad Garcia, Angelique F. Ralph, Supreet Saluja and Eve T. House. Writing – review and editing: All authors.

## Funding

The roundtable meeting was funded by the Eating Disorders In weight‐related Therapy Collaboration, funded by National Health and Medical Research Council Grant #2002310. Hiba Jebeile receives salary support through award of an NHMRC Emerging Leadership Investigator Grant #2017139. Funding sources had no role in the planning, meeting, writing or publication of the work.

## Disclosure

Not commissioned; externally peer reviewed.

## Conflicts of Interest

Hiba Jebeile is an advisor for The Obesity Collective and is on the leadership team of the Health Behaviour and Weight Management Interest Group of Dietitians Australia (both unpaid). Milan K. Piya has received honoraria from Novo Nordisk, Eli Lilly, Johnson and Johnson and Boehringer Ingelheim for advisory boards and speaking. He is the vice president of the Australian and New Zealand Obesity Society and a member of the Diabetes and Endocrinology Group, Agency for Clinical Innovation (NSW Government). Phillipa Hay has received sessional fees from the Australian Medical Council and the Health Education and Training Institute, and royalties and honoraria from Hogrefe and Huber, McGraw Hill Education and BioMed Central. She has prepared a report under contract for Takeda Pharmaceuticals (formerly Shire) regarding binge eating disorder (July 2017), was a consultant to Takeda Pharmaceuticals and is a consultant to Tryptamine Therapeutics. She is the chair of the steering committee of the National Eating Disorders Collaboration. Evan Atlantis holds an honorary position as the secretary of the National Association of Clinical Obesity Services. Leah Brennan is a member of the steering committee of the National Eating Disorders Collaboration and director of the Centre for Eating, Weight and Body Image. Sarah Trobe is the director of the National Eating Disorders Collaboration. Natalie B. Lister is the chair of the Australia New Zealand Paediatric Obesity Network and a committee member of the Sydney Regional Group of the Nutrition Society of Australia.

## Supporting information


**Data S1:** Supplementary figures and tables.

## Data Availability

Data are published in this article.
