# Supplementary material for: Progressing Cross‐Sector Collaboration for People With Eating Disorders and Higher Weight: Priority Actions From an Expert Roundtable Using a Modified Nominal Group Technique
Source: Med J Aust. 2026 Jul 1;224(7):e70235. doi: 10.5694/mja2.70235 (PMC13323596; doi:10.5694/mja2.70235)
Supplement: Supplementary file 1 — Data S1: Supplementary figures and tables. [file MJA2-224-0-s001.pdf]

MJA
The Medical Journal of Australia

Supporting Information

Supplementary material

This appendix was part of the submitted manuscript and has been peer reviewed.
It is posted as supplied by the authors.

Appendix to: Jebeile H, Brennan L, Burrows T, et al. Progressing Cross-Sector Collaboration for People With Eating Disorders and Higher Weight: Priority Actions From an Expert Roundtable Using a Modified Nominal Group Technique. *Med J Aust* 2026; doi: 10.5694/mja2.70235.

Table S1: Key issues and possible solutions within the NEDC Stepped System of Care Framework

BARRIERS AND ISSUES	SOLUTIONS, CURRENT PROGRAMS, GAPS
**PREVENTION**	
Potentially harmful messaging and language used to discuss food, nutrition, and bodies in public health campaigns, social media, education settings, within the fitness industry and within families	• Develop and implement public health campaigns, language guides and social media standards based on NEDC Eating Disorder Safe Principles<br>• Research identifying protective social media communication strategies<br>• Improve health literacy across all settings<br>• Investment in First Nations led research into ED prevention and experiences such as body shame, food shame and lifestyle stigma
Lack of awareness of the co-occurrence of eating disorders and higher weight, among health workforce, media, community and parents/carers.<br>Lack of mechanistic understanding of the co-occurrence of eating disorders and higher weight	• Funding for development and roll-out of dual prevention programs<br>• Promote whole of community understanding that ED occurs in people with higher weight e.g. via social media campaigns<br>• Research on development pathways for eating disorders and higher weight
Limited workforce capacity to implement evidence-based prevention approaches in schools and universities	• Engagement with schools to co-design and deliver evidence-based prevention programs<br>• Advocacy for government investment in implementation of existing ED prevention programs. e.g., Butterfly Foundation, in schools.<br>• Extension of existing mental health literacy programs to encompass EDs<br>• Research examining efficacy of existing programs to inform further roll-out<br>• Research into dietitian/nutritionist-delivered school nutrition curriculum<br>• School-based prevention programs that address weight stigma and appearance-based bullying
Limited workforce capacity and absence of a system of care that is sensitive to the needs of high-risk groups (e.g. people with a history of trauma, children and adolescents, peri-menopausal women, people with multiple co-morbidities)	• Advocate for the importance of trauma prevention program (i.e., preventing family violence, child abuse), inclusion, culturally safe environments, and intersectional approaches<br>• Upskill workforce on needs of high-risk groups
Limited recognition of the impact of the food environment and social determinants of health on engagement in health behaviours and their influence on mental health	• Improvements to the food environment e.g., eradication of food desserts, food marketing reform<br>• Development of regulatory, economic and social strategies to better address food security as a risk factor for eating disorders and higher weight

Weight Stigma is prevalent across all corners of society, within and beyond healthcare in images, physical environment, and language.	• Expansion of discrimination legislation to include weight-based discrimination<br>• Investment in research regarding weight stigma to increase understanding of prevalence and drivers of weight stigma<br>• Heighten health professional and community understanding of weight stigma through training and public health campaigns<br>• Family-based education on weight-related communication and bullying
**IDENTIFICATION**	
Lack of health professional and community recognition of and harmful stereotypes regarding the co-occurrence of eating disorders and higher weight	• Educate health professionals on identifying EDs in people with higher weight, clustering with other health conditions e.g. depression and anxiety, available treatment pathways, and making appropriate referrals<br>• Disseminate education via existing education pathways e.g. NEDC, PHNs, NACOS, EDIT, RACGP, InsideOut Institute GP Hub, CoRe-ED<br>• Adopt mental health first aid guidelines to support ED identification and training for the community<br>• Support parent understanding of disordered eating behaviours across the weight spectrum<br>• Ensure an understanding of scope of practice concerning the identification of ED/high weight outside of health settings e.g. schools, community
Lack of health professional awareness of best practices for discussing and assessing weight-related risk and eating behaviours	• Ensure clear scope of practice to promote confidence in understanding duty of care<br>• Promote safe and compassionate care during communication about weight and eating behaviours that is patient-centred, and trauma informed<br>• Provide guidance on when, how, and whether to have a discussion about weight and/or eating behaviours in line with patient-centred care<br>• Educate health professionals (across primary care and specialist services) to provide early and opportunistic screening for weight-related health risks and eating disorders e.g. using EOSS-2/EOSS-P and eating disorder screening tools
Many existing eating disorder screening tools were informed by an understanding of anorexia nervosa and restraint theory and have limited utility for people with higher weight (including unclear validation and norms)	• Research to validate existing and develop new screening and assessment methods for identifying all eating disorders in people with higher weight, including different populations (varying age, gender, literacy level, high weight and clinical obesity), context and treatment settings (including in the absence of a psychologist), with low-cost digital implementation
Lack of consistent screening and assessment procedures across weight management, mental health,	• Establish a structured protocol to support consistent approaches to eating disorder screening and assessment<br>• Establish 'champions' in professional education/training settings

bariatric surgery and other health settings where people seek care	
Limited public awareness of support pathways	• Promote public awareness of referral pathways, support consumers to navigate the system<br>• Enhance support for self-identification and advocacy tools e.g. online navigation tool
Weight stigma contributes to the development of ED and can impede their identification in people with higher weight, may hinder diagnosis of clinical obesity and may prevent engagement with recommended referral pathways	• Train health professionals to recognise and address internalised stigma and promote using a compassionate approach and openly listening to patient concerns<br>• Safe, accessible, and welcoming health services e.g. appropriate seating, imagery, language, equipment
**INITIAL RESPONSE**	
Lack of resources to support families in the time between identification and engagement with services and those with subthreshold disordered eating behaviours	• Develop and disseminate technology-enabled self-help resources (e.g., AI chatbots and online guided self-help) and family/peer support materials to bridge the care gap<br>• Increase awareness and dissemination of existing resources (including navigating pathways)
Limited capacity of the primary healthcare system to respond due to limited time, lack of staff confidence, and lack of MBS items	• Provide education to support better utilisation of ED management plans e.g. via PHNs<br>• Advocate for additional MBS items to allow for longer appointments for GP assessment at low-cost to consumer
Limited referral pathways and ongoing follow-up, including lack of access to tertiary weight management programs and missing system to monitor outcomes of initial response	• Establish referral pathways appropriate for people with higher weight across the spectrum of care<br>• Enhance health professional awareness of treatment pathways following screening, including patient self-advocacy tools<br>• Advocate for additional services by demonstrating the impact of comorbid ED/higher weight on overall health
Lack of tools and models of care to support First Nations, culturally diverse and neurodiverse communities	• Prioritise the development of neuro-inclusive, culturally safe care environments<br>• Initial response is provided in a safe and culturally appropriate way
**TREATMENT: PRIMARY, SECONDARY, TERTIARY**	
Lack of collaborative and personalised care models addressing eating disorders and higher weight	• Collaborative care models between ED and obesity services with integrated case conferencing and co-location of services to ensure consistent messaging and goals across the treating team<br>• Establish consensus between the fields regarding evidence and research priorities to inform treatment approaches<br>• Research to inform understanding of long-term outcomes of siloed treatment

	• Research to inform models of care (with lived experience involvement) to develop personalised, trauma-informed co-treatment approaches
Lack of support for patients to develop awareness of treatment options and be involved in treatment decisions	• Embed care coordination in treatment services<br>• Patient education on possible outcomes of treatments
High-cost and limited treatment services (particularly in the public system e.g., low weight criteria for eating disorder services, long waiting lists, and in regional areas), which are inadequately covered under existing Medicare rebates	• Investment in affordable and accessible treatment options, providing services (including telehealth/online, self and clinician-led services) that bridge the gap between primary care and specialist services<br>• Establish linkage between treatment services and Aboriginal medical services<br>• Increased Medicare funding to access ED and weight management services<br>• Research examining cost-effectiveness of evidence-based treatments
Limited provider knowledge of ED and higher weight management, which may lead to insufficient treatment options presented to patients and inappropriate treatment goals (e.g. singular focus on weight loss)	• Develop and disseminate interdisciplinary provider training on co-treatment, patient-centred and trauma-informed care, and weight-inclusive language through professional organisations and as part of higher education e.g., medical training<br>• Develop a credentialing system for ED and higher weight training, including weight stigma, and increase availability of supervision for health professionals providing care for ED and higher weight
Experiences of weight stigma in treatment settings e.g. inappropriate language leading to feelings of blame/shame, imagery, accessibility issues	• Training on weight-related language and weight stigma across the treatment workforce<br>• Train health professionals to recognise and address internalised stigma<br>• Create safe, accessible, and welcoming health services e.g. appropriate seating, imagery, equipment<br>• Provision of online and telehealth options to help prevent stigma
Additional complexities in specialist settings and circumstances e.g. in bariatric surgery ED assessment is not a routine part of care in the private sector and disclosure of ED may be perceived to prevent access to care; in fertility care language used can often pathologise bodies	• Collaborate with ANZMOSS to implement training for bariatric surgeons on ED assessment, language use, and a holistic approach to care.<br>• Investment in increased access to bariatric surgery in the public health system
**PSYCHOSOCIAL AND RECOVERY SUPPORT**	
Limited support for care navigation, including getting to appointments, accessing online treatment and coordinating ongoing appointments.	• Engagement with the community sector to improve care navigation support<br>• Support holistic care recognising the hierarchy of needs e.g. housing, safety.

Limited financial support to access ongoing care across mental health and medical settings, limiting access to MDTs in the community due to the requirement to pay for multiple services	• Research to explore novel models of long-term care (e.g. using AI) and to demonstrate the benefits and cost-effectiveness of long-term care<br>• Development of self-help resources, telehealth and online support/programs to support long term care<br>• Advocate for expanded Medicare and NDIS funding to support long-term, multidisciplinary care
Limited support with relapse prevention	• Build a peer workforce, linked to existing groups (e.g., Weight Issues Network), focused on supporting people with co-occurring ED and higher weight<br>• Normalise the cyclical nature of treatments

ACCORD Checklist

Item No.	Section	Checklist Item (*help text*)	Page No.
T1	**Title**	Identify the article as reporting a consensus exercise and state the consensus methods used in the title.<br>*For example, Delphi or nominal group technique.*	1
I1	**Introduction**	Explain why a consensus exercise was chosen over other approaches.	2
I2		State the aim of the consensus exercise, including its intended audience and geographical scope (national, regional, global).	2
I3		If the consensus exercise is an update of an existing document, state why an update is needed, and provide the citation for the original document.	NA
M1	**Methods**<br>Registration	If the study or study protocol was prospectively registered, state the registration platform and provide a link. If the exercise was not registered, this should be stated.<br>*Recommended to include the date of registration.*	Not registered
M2	Selection of SC and/or panellists	Describe the role(s) and areas of expertise or experience of those directing the consensus exercise.<br>*For example, whether the project was led by a chair, co-chairs or a steering committee, and, if so, how they were chosen. List their names if appropriate, and whether there were any subgroups for individual steps in the process.*	Supplementary file
M3		Explain the criteria for panellist inclusion and the rationale for panellist numbers. State who was responsible for panellist selection.	3<br>Supplementary file
M4		Describe the recruitment process (how panellists were invited to participate).<br>*Include communication/advertisement method(s) and locations, numbers of invitations sent, and whether there was centralised oversight of invitations or if panellists were asked/allowed to suggest other members of the panel.*	Supplementary file
M5		Describe the role of any members of the public, patients or carers in the different steps of the study.	3<br>Supplementary file
M6	Preparatory research	Describe how information was obtained prior to generating items or other materials used during the consensus exercise.<br>*This might include a literature review, interviews, surveys, or another process.*	3<br>Supplementary file
M7		Describe any systematic literature search in detail, including the search strategy and dates of search or the citation if published already.<br>*Provide the details suggested by the reporting guideline PRISMA and the related PRISMA-Search extension.*	NA
M8		Describe how any existing scientific evidence was summarised and if this evidence was provided to the panellists.	Supplementary file
M9	Assessing consensus	Describe the methods used and steps taken to gather panellist input and reach consensus (for example, Delphi, RAND-UCLA, nominal group technique).<br>*If modifications were made to the method in its original form, provide a detailed explanation of how the method was adjusted and why this was necessary for the purpose of your consensus-based study.*	3<br>Supplementary file
M10		Describe how each question or statement was presented and the response options. State whether panellists were able to or required to explain their responses, and whether they could propose new items.<br>*Where possible, present the questionnaire or list of statements as supplementary material.*	Supplementary file
M11		State the objective of each consensus step.<br>*A step could be a consensus meeting, a discussion or interview session, or a Delphi round.*	3<br>Supplementary file
M12		State the definition of consensus (for example, number, percentage, or categorical rating, such as 'agree' or 'strongly agree') and explain the rationale for that definition.	Supplementary file
M13		State whether items that met the prespecified definition of consensus were included in any subsequent voting rounds.	N/A
M14		For each step, describe how responses were collected, and whether responses were collected in a group setting or individually.	Supplementary file
M15		Describe how responses were processed and/or synthesised.<br>*Include qualitative analyses of free-text responses (for example, thematic, content or cluster analysis) and/or quantitative analytical methods, if used.*	3<br>Supplementary file

M16		Describe any piloting of the study materials and/or survey instruments.<br>*Include how many individuals piloted the study materials, the rationale for the selection of those individuals, any changes made as a result and whether their responses were used in the calculation of the final consensus. If no pilot was conducted, this should be stated.*	N/A
M17		If applicable, describe how feedback was provided to panellists at the end of each consensus step or meeting.<br>*State whether feedback was quantitative (for example, approval rates per topic/item) and/or qualitative (for example, comments, or lists of approved items), and whether it was anonymised.*	Supplementary file
M18		State whether anonymity was planned in the study design. Explain where and to whom it was applied and what methods were used to guarantee anonymity.	N/A
M19		State if the steering committee was involved in the decisions made by the consensus panel.<br>*For example, whether the steering committee or those managing consensus also had voting rights.*	Supplementary file
M20	Participation	Describe any incentives used to encourage responses or participation in the consensus process.<br>*For example, were invitations to participate reiterated, or were participants reimbursed for their time.*	Supplementary file
M21		Describe any adaptations to make the surveys/meetings more accessible.<br>*For example, the languages in which the surveys/meetings were conducted and whether translations or plain language summaries were available.*	N/A
R1	Results	State when the consensus exercise was conducted. List the date of initiation and the time taken to complete each consensus step, analysis, and any extensions or delays in the analysis.	2
R2		Explain any deviations from the study protocol, and why these were necessary.<br>*For example, addition of panel members during the exercise, number of consensus steps, stopping criteria; report the step(s) in which this occurred.*	N/A
R3		For each step, report quantitative (number of panellists, response rate) and qualitative (relevant socio-demographics) data to describe the participating panellists.	N/A
R4		Report the final outcome of the consensus process as qualitative (for example, aggregated themes from comments) and/or quantitative (for example, summary statistics, score means, medians and/or ranges) data.	Table 1<br>Figure 1
R5		List any items or topics that were modified or removed during the consensus process. Include why and when in the process they were modified or removed.	N/A
D1	Discussion	Discuss the methodological strengths and limitations of the consensus exercise.<br>*Include factors that may have impacted the decisions (for example, response rates, representativeness of the panel, potential for feedback during consensus to bias responses, potential impact of any non-anonymised interactions).*	Supplementary file
D2		Discuss whether the recommendations are consistent with any pre-existing literature and, if not, propose reasons why this process may have arrived at alternative conclusions.	3-9
O1	Other information	List any endorsing organisations involved and their role.	NA
O2		State any potential conflicts of interests, including among those directing the consensus study and panellists. Describe how conflicts of interest were managed.	Supplementary file
O3		State any funding received and the role of the funder.<br>*Specify, for example, any funder involvement in the study concept/design, participation in the steering committee, conducting the consensus process, funding of any medical writing support. This could be disclosed in the methods or in the relevant transparency section of the manuscript. Where a funder did not play a role in the process or influence the decisions reached, this should be specified.*	Title page

From: PLoS Med 21(1): e1004326. https://doi.org/10.1371/journal.pmed.1004326 For more information see: https://www.ismpp.org/accord

Supplementary file: Progressing cross-sector collaboration for people with eating disorders and higher weight: Priority actions from an expert roundtable

Additional methods

Planning

Committee members

Member	Affiliation
Dr Sarah Trobe (Chair)	National Director, National Eating Disorders Collaboration<br>Clinical Psychologist
Dr Hiba Jebeile	NHMRC Emerging Leadership Fellow, EDIT Collaboration, The University of Sydney
Prof Louise A. Baur	Professor of Paediatrics & Child Health, The University of Sydney<br>Consultant Paediatrician, Weight Management Services, The Children's Hospital at Westmead<br>Co-Chair, NHMRC Guidelines for the treatment of obesity
Prof John Dixon	Vice President, National Association of Clinical Obesity Services<br>General Practitioner
Prof Tim Gill	Director, The Obesity Collective
Prof Phillipa Hay	Professor and Chair of Mental Health, Western Sydney University<br>Psychiatrist and SWSLHD Director Mental Health Research, Camden and Campbelltown Hospitals, SWSLHD, NSW<br>Chair, NEDC Guidelines working group for the treatment of eating disorders and higher weight<br>Chair of Steering Committee, National Eating Disorders Collaboration
A/Prof Samantha Hocking	President, National Association of Clinical Obesity Services<br>Endocrinologist, Metabolic Obesity Service, Royal Prince Alfred Hospital, SLHD, NSW
Dr Angelique Ralph	Research Lead, National Eating Disorders Collaboration<br>Australian Catholic University

Declaration of interests of committee members who participated in the consensus process

ST, HJ, TG, AR have no potential conflicts to declare.

LAB: Speakers bureau, Novo Nordisk and Eli-Lilly. Funds directed back to my institutional research cost centre. Novo Nordisk sponsored the ACTION Teens Trial, a multi-country on-

line survey of the attitudes, behaviours and barriers for obesity care in adolescents with obesity, their caregivers and health professionals. LAB was the principal investigator of the Australia arm of the study.

PH: Received sessional fees from the Australian Medical Council, Health Education and Training Institute (HETI, NSW), and royalties/honoraria from Hogrefe and Huber, McGraw Hill Education and BioMed Central. She has prepared a report under contract for Takeda (formerly Shire) Pharmaceuticals regarding binge eating disorder (July 2017), and was a consultant to Takeda Pharmaceuticals and is a consultant to Tryptamine Pharmaceuticals. She is co-Editor in Chief Journal of Eating Disorders.

Role

A Committee was formed in June 2024 to discuss how to bridge the divide between the fields of obesity and eating disorders. The Committee acknowledged the complexity of these intersecting areas but identified common ground in supporting patients experiencing both eating disorders and higher weight. It was agreed that an in-person roundtable meeting was needed to bring people together across fields, create a safe space to have productive conversations on eating disorders and higher weight, and identify shared priority areas for future work together.

The Committee played a central role in overseeing the planning and delivery of the roundtable meeting and refined its purpose and scope. Members identified and recommended potential attendees to ensure appropriate expertise and representation across fields, role, and profession.ST and HJ then led the planning, invitation of participants and conduct of the meeting. Invited participants were asked what they hoped to achieve from the day and the Committee then developed the agenda to support focused and productive discussion to address those priorities.

Attendee selection and invitation

Possible roundtable participants were purposively identified by the Committee to ensure diverse representation of multidisciplinary researchers and clinicians working in the fields of obesity, eating disorders or both, and people with lived experience of both conditions. Potential participants (n=38) were contact via email through an expression of interest process (see email below). Invitations were for the identified participants and could not be transferred to another person. Invited participants could suggest additional people for consideration by the committee. Some members of the committee attended and participated in the roundtable meeting.

Participants did not receive financial incentives to attend the meeting. Some travel support was provided to attendees on a case-by-case basis including for lived experience attendees. No funding was provided by external industry or organisations with competing interests.

Expression of interest email

Dear [invited participant],

The Eating Disorders in Weight-Related Therapy (EDIT) Collaboration and the National Eating Disorders Collaboration (NEDC) are organising a round table discussion in November to explore existing work and activities, commonalities and shared goals between the eating disorder and high weight/obesity sectors and identify opportunities for collaborations. We anticipate between 20-25 people will attend this meeting, representing clinical, lived experience, and research roles from within and across the eating disorder and weight/obesity sectors.

We warmly invite you to join us for this initial meeting.

To determine the best location and format for the meeting, we seek your feedback on these questions:

- Would you be interested in joining the first roundtable discussion with EDIT and NEDC? YES/NO
- Would you be able to attend an (approximate) 4-hour meeting on either Friday 22 November or Friday 29 November (please indicate your availability for each date)
    - Friday 22 November 2024 - YES/NO
    - Friday 29 November 2024 - YES/NO
- Would you be able to attend the meeting in Sydney or Melbourne (please indicate your preference)? SYDNEY/MELBOURNE
- If you are unable to attend in Sydney or Melbourne, would you be able to attend online? YES/NO
- In your view, what would be the most important outcomes for this meeting?

If you would like any further information about this work and planned meeting, please don’t hesitate to reach out to me and we can organise a time to speak.

Kind regards,
Dr Hiba Jebeile, EDIT Collaboration
Dr Sarah Trobe, NEDC National Manager

Roundtable meeting

An in-person roundtable meeting was held on 29th November 2024 at The Charles Perkins Cetnre, The University of Sydney, Australia.

Aim

Based on the feedback from participants, the aim of the roundtable meeting was to establish collaborations and relationships across fields, and to develop shared goals and priority actions.

Facilitators

Dr Hiba Jebeile, Co-Lead, EDIT Collaboration, The University of Sydney

Dr Hiba Jebeile is an NHMRC Emerging Leadership Fellow and Accredited Practicing Dietitian at The University of Sydney, Australia. As a dietitian she has provided community-based obesity programs for children and families, chronic disease management and supported clinical trials. Hiba's current research explores the intersection between behavioural weight management interventions and change in eating disorder symptoms, and screening protocols for the early identification of eating disorders in people with higher weight. She is Co-lead and Program Manager of the Eating Disorders In weight-related Therapy (EDIT) Collaboration, a world-first international collaboration of researchers, clinicians and people with lived experience exploring individual variability in eating disorder risk during behavioural interventions.

Dr Sarah Trobe, National Director, National Eating Disorder Collaboration

Dr Sarah Trobe is the National Director of the National Eating Disorder Collaboration, leading a team which develops and implements consistent national standards for the prevention and treatment of eating disorders. Sarah is a Clinical Psychologist and has held clinical positions at the Royal Children's Hospital CAMHS and Weight Management Service and private practice as well as working with The Victorian Centre of Excellence in Eating Disorders (CEED) with a focus on service development, case consultation, multidisciplinary care coordination, and education and training provision. Sarah's interests lie in the link between physical health and mental health and current care structures in this field, and she is passionate about setting up strong treatment networks and systems across the country. Sarah guides and supports all of NEDC's work, is leading the implementation of the National Eating Disorders Strategy 2023-2033, and provides expertise into workforce development initiatives.

Pre-reading

Prior to the meeting, participants were sent the following pre-reading documents:

A Centre of Translation Research and Action for Eating and Weight Disorders (ASTRA) (2018) White paper.

Da Luz FQ, Hay P, Touyz S, Sainsbury A. Obesity with comorbid eating disorders: associated health risks and treatment approaches. Nutrients. 2018 Jun 27;10(7):829

Agenda
The agenda for the meeting is in Appendix A.

Introduction
The introduction outlined the focus of the meeting to be specifically on the intersection of eating disorders and clinical obesity, with an effort to remain within this scope while acknowledging there may be moments of broader discussion. It was highlighted that the preferred language used to discuss weigh may vary among participants, and establishing shared terms for the purpose of the session. The introduction also focused on creating a safe and open space grounded in curiosity, mutual respect, and openness to different perspectives from the diverse range of participants present.

Participants were invited to introduce themselves, describe their professional role, and outline their interests, affiliations, positions, experience and perspectives relating to obesity and eating disorders. The meeting included individuals representing differing professional perspectives and these divergent perspectives were acknowledged and managed respectfully to support constructive discussion.

Setting the scene
A series presentations were given to set the scene and summarise current evidence and areas of intersection across eating disorders and clinical obesity.

- Clinical obesity - Prof Louise Baur
- Neurobiology - Prof John Dixon
- Eating disorders - Prof Phillipa Hay
- Prevalence - Ms Hannah Melville
- Outline the issue - Prof Phillipa Hay
- Lived experience perspectives – three presentations from people with lived experience were shared

Consensus activities
A modified Nominal Group Technique (NGT) was used for the one-day meeting to facilitate structured idea generation, discussion and prioritisation. Participants independently generated ideas to key questions introduced by facilitators. This was followed by facilitator-led group discussion to summarise, clarify and refine ideas. Participants were then invited to individually identify priority items. These were tallied and discussed as a group to reach consensus.

Step 1: Understanding the problem
Objective: Understand the problems and start to generate solutions

For each component of the NEDC Stepped System of Care (Appendix B), roundtable

participants were asked to identify issues and barriers that would affect outcomes for people with higher weight and eating disorders.

Attendees were asked to write down on sticky notes as many current problems, barriers, issues they could identify. These were posted and organised on the wall according to the NEDC Stepped System of Care Framework (E.g., research lacking in treatment, need for prevention programs, need for health service re-design to support both issues, upskilling all workforces):

1. Prevention
2. Identification
3. Initial Response
4. Treatment: primary, secondary, tertiary
5. Psychosocial and recovery support

Synthesis of step 1: During the lunch break facilitators, with the support of NEDC and EDIT staff, organised sticky notes into themes within the Stepped System of Care and these were summarised on PowerPoint slides. Following the break and as a large group, facilitators then summarised the key themes within each segment of the Stepped System of Care framework and facilitated a discussion to identify any major omissions or errors.

Step 2: What is being done and what could be done – possible actions

Objective: Outline the existing and possible initiatives across clinical, research, advocacy and public health.

To address each issue and barrier identified in Step 1, participants were asked to identify: 1) Existing programs, research and initiatives that were addressing the issue; and 2) Opportunities for new or expansion of existing initiatives, and to write these onto sticky notes and place them alongside each issue. Participants were asked to move through each of the themes and consider all issues/barriers.

Step 3: Identifying priority actions

Objective: To prioritise the identified opportunities and actions

Each participant was provided with five coloured dots and was asked to identify their top five priority areas of action from those identified in Step 2 by placing a coloured dot next to that initiative. Participants could add multiple dots to one item or spread these across initiatives.

Synthesis of step 3: During the afternoon break, coloured dots were tallied by facilitators and the top six priority areas for action were identified.

Step 4: Consolidation

Objective: Achieve agreement on the priority areas for action

The facilitators presented the six identified priority areas, and the group discussed these to

ensure they did represent the most important actions needed to improve outcomes, and to identify any potential disagreement. This was followed by a discussion on what would be needed to achieve success and the next steps following the Roundtable. It was raised that to achieve these priorities, a safe space to discuss and collaborate across these intersecting areas would be needed. It was proposed and agreed that working groups, led by NEDC/EDIT, would be formed to progress each of the priority areas.

Post-meeting

Following the meeting, minutes were circulated to all invited participants with a summary of the identified issues, solutions and priority actions. Participants were provided with the opportunity to provide additional feedback following the event by email and to comment on the summarised outcomes of the meeting.

Strengths and limitations

Strengths of this process included balanced representation across the fields of obesity and eating disorders and across disciplines, ensuring a breadth of perspectives informed the discussion. The meeting aims were shaped through an expression-of-interest process and participant-led identification of expected outcomes. The modified nominal group technique approach provided dedicated time for both individual idea generation and group discussion. The open and inclusive format allowed all attendees to contribute ideas without restriction. This openness encouraged broader perspectives to be shared. Regularly scheduled breaks also facilitated relationship building and collaboration among participants, supporting open discussion and effective information sharing across this often-sensitive area.

Several limitations should also be considered. Although the nominal group technique was used to promote equitable participation, it is possible that more confident or vocal individuals contributed disproportionately to group discussions. The lack of anonymity inherent in in-person meetings may also have constrained the expression of more sensitive or opposing perspectives. Participants were offered an opportunity to provide further input via email after the meeting.

Conflicts of interest

All participants were invited on the basis of their recognised expertise in the field. At the commencement of the meeting, all participants were asked to introduce themselves, describe their professional role, and outline their interests and perspectives relating to obesity and eating disorders. Participants were also invited to declare any relevant interests, affiliations, or positions that may influence their contributions.

The meeting included individuals representing differing professional perspectives, including those involved in weight-loss–oriented interventions and those practising weight-neutral approaches. Divergent perspectives were acknowledged and managed respectfully to support constructive discussion.

Presenters were asked to declare potential conflicts of interest at the beginning of their talk.

Appendix A: Roundtable meeting agenda

National Eating Disorders Collaboration — Evidence | Experience | Expertise

EDIT Collaboration — Eating Disorders In weight-related Therapy

Agenda

NEDC & EDIT Roundtable

**Date:**	Friday 29 November 2024
**Time:**	**10:00am – 4:00pm AEDT (Melb/Sydney), Arrival from 9.30am**
**Venue:**	The Mackenzie Room, Level 6, Charles Perkins Centre, John Hopkins Drive, The University of Sydney
**Meeting chair:**	Sarah Trobe & Hiba Jebeile
**Pre-reading**	A Centre of Translation Research and Action for Eating and Weight Disorders (ASTRA) (2018) White paper.<br>Felipe Q. da Luz, Phillipa Hay, Stephen Touyz, and Amanda Sainsbury (2018) Obesity with Comorbid Eating Disorders: Associated Health Risks and Treatment Approaches. Nutrients, 10, 829. Accessed at https://pmc.ncbi.nlm.nih.gov/articles/PMC6073367/

	**Agenda topics**
**10:00am**	**Welcome** (Sarah Trobe and Hiba Jebeile)
**10:10am**	**Introductions** (Hiba Jebeile)
**10:50am**	**Defining the issue**<br>• Clinical obesity: Louise Baur<br>• Neurobiology: John Dixon<br>• Eating disorders: Phillipa Hay<br>• Prevalence: Hannah Melville<br>• Outlining the issue: Phillipa Hay
**11:30am**	**Lived experience perspectives** – three speakers
**11:50am**	**Activity: Understanding the problem** (Sarah Trobe)
**12:15pm**	**LUNCH**

**1:00pm**	**Feedback from activity** (Hiba Jebeile)
**1:30pm**	**Activity: What is being done and what could be done?** (Sarah Trobe)
**2.10pm**	**Activity: Identifying priority actions** (Hiba Jebeile)
**2:30pm**	**BREAK**
**2:50pm**	**Creating change – actions, barriers, and outcomes** (Sarah Trobe)
**3:30pm**	**Next steps** (Hiba Jebeile)<br>• Working groups<br>• Ongoing collaboration<br>• Representation
**4:00pm**	**Close**

Appendix B: NEDC Stepped System of Care Framework

NEDC Stepped System of Care Framework includes five components: 1. *Prevention* refers to actions, programs, or policies that aim to reduce modifiable risk factors, and/or bolster protective factors; 2. *Identification* refers to the detection of warning signs or symptoms, and engagement with the person who may be experiencing an eating disorder and higher weight, to support access to an initial response; 3. *Initial Response* comprises an initial assessment and preliminary diagnosis, and referral to the most appropriate treatment options based on the person's psychological, physical, nutritional, and psychosocial needs; 4. *Treatment* (primary, secondary, tertiary) refers to the primary intervention for eating disorders and/or clinical obesity; and 5. *Psychosocial and Recovery Support* refers to services and programs which support broader psychological and social needs of the person, and support a person experiencing an eating disorder to engage with or sustain recovery or improved quality of life and assist family/supports and community in their caring role.

Appendix C: Eating Disorder Safe Principles

The Eating Disorder Safe (ED Safe) principles are a policy and practice framework aimed at embedding eating disorder prevention and harm minimisation across multiple arenas. They were developed by NEDC in 2024 as a key output of the National Eating Disorders Strategy 2023-33. Centring around five key domains – health, food, mind, body and harm minimisation – the principles set out key considerations for reducing eating disorder risk and harm which can be applied at the individual, group, or population level. Crucially, First Nations Perspectives are embedded within the ED Safe principles and ought also to be in all implementation activities. The 19 Eating Disorder Safe Principles are:

Health

- Lifespan approaches: Families are supported to experience and model positive relationships with food, bodies and movement from the earliest opportunity and across the lifespan.
- Disability and chronic health conditions: The unique relationships between eating, chronic illness, disability and experiences of healthcare are acknowledged and addressed.
- Inclusive communication: Communications use size-inclusive, non-stigmatising language and imagery in respect of health, food, minds and bodies.
- Movement: People are supported to experience movement and exercise in ways that promote their overall wellbeing, including their emotions, social connections, and physical and mental health.

Food

- Neutral food language: Neutral language is used to describe food and its properties and food messaging is appropriate for its audience.
- Dietary advice: Dietary advice is given in a weight neutral, culturally and developmentally appropriate manner, by people with appropriate qualifications and experience according to their scope of practice.
- Food security: Food security is addressed to support safe relationships with food and eating at individual and population levels.
- Diet culture: Diet culture and its far-reaching consequences are understood, reduced and ultimately eliminated, with actions based on contemporary evidence.

Mind

- Mental health stigma: Mental health stigma and its harms in relation to health, food, mind and body are of concern and must be eliminated.
- Relationship between mental health and food: The bidirectional relationship between food, eating and mental health is recognised, within the context of individual, social, cultural and economic factors.

Body

- Inclusive environments: Settings where people live, learn, work, play and receive help are inclusive and welcoming for people of all sizes, shapes, weights, genders, sexualities, cultures, neurotypes and abilities.
- Neutral language about bodies: Neutral language is used to describe bodies at all ages and stages of development, all sizes, shapes, abilities and ethnicities.
- Non-discrimination: Size, shape or weight are not used as a standalone measure of health or wellbeing, used as a measure of skill, aptitude or performance, or a barrier to education, employment or civic participation.
- Weight stigma: Weight stigma and its consequences are of concern and must be eliminated.
- Systemic factors: System-level drivers of eating disorder risk, for example institutional weight stigma, diet culture, medical and health professional weight bias, are addressed.

Harm minimisation

- Alternative metrics: Alternatives to weight-focused activities and outcomes are prioritised as far as possible (e.g., other health, quality of life, performance, and wellbeing metrics).
- Identification and response: Recognising and responding to eating disorder warning signs and symptoms in all people and bodies is a responsibility of all people working in high-risk settings.
- Weight stigma in health: Weight stigma has no place in the healthcare setting, no matter the condition being treated.
- Risk management: Eating disorder risk is a consideration when making decisions around a person's health, care, learning, work or sports participation

Resources to support the translation of ED Safe principles into practice across a wide range of settings are available. Further information on the Stepped System of Care or Eating Disorder Safe Principles can be found at www.nedc.com.au.
